# Albumin-corrected anion gap and short-term mortality in patients with sepsis and heart failure: A retrospective cohort study

**DOI:** 10.1097/MD.0000000000047383

**Published:** 2026-01-30

**Authors:** Qiao Xiao, Tingting Liu, Xinyuan Zhang, Zhiling Sun

**Affiliations:** a Department of Cardiology, Affiliated Hospital of Nanjing University of Chinese Medicine, Jiangsu Provincial Hospital of Chinese Medicine, Nanjing, China; b Department of Critical Care Medicine, Affiliated Hospital of Nanjing University of Chinese Medicine, Jiangsu Provincial Hospital of Chinese Medicine, Nanjing, China; c School of Nursing, Nanjing University of Chinese Medicine, Nanjing, China.

**Keywords:** albumin-corrected anion gap, heart failure, mortality, prognosis, risk stratification, sepsis

## Abstract

The conventional anion gap (AG) is widely used to evaluate metabolic acidosis, but its accuracy is limited in hypoalbuminemia, a common condition among critically ill patients. Albumin-corrected anion gap (ACAG) has been proposed to overcome this limitation, yet its prognostic value in patients with sepsis complicated by heart failure (HF) remains unclear. We retrospectively analyzed adult patients with sepsis and HF from the Medical Information Mart for Intensive Care IV database. ACAG was calculated as (44 – serum albumin [g/L]) × 0.25 + AG, and patients were stratified into high (≥20 mmol/L) and low (<20 mmol/L) groups using X-tile–derived thresholds. The primary outcomes were 28-day and in-hospital mortality. Survival was assessed using Kaplan–Meier curves with log-rank tests. Independent associations were examined with multivariable Cox regression. Restricted cubic splines were applied to evaluate nonlinear trends. Variable importance was assessed using the Boruta algorithm. Discrimination between ACAG and AG was compared using receiver operating characteristic curves and DeLong’s test. Subgroup analyses explored consistency across clinical strata. A total of 715 patients were included, of whom 366 were classified into the high-ACAG group. High ACAG was associated with significantly higher 28-day and in-hospital mortality (log-rank *P* < .001). Multivariable Cox regression confirmed ACAG as an independent risk factor for poor prognosis (28-day mortality: hazard ratio = 1.80, 95% confidence interval: 1.27–2.56; in-hospital mortality: hazard ratio = 1.75, 95% confidence interval: 1.20–2.54). Restricted Cubic Splines showed a nonlinear association with 28-day mortality and a near-linear relationship with in-hospital mortality. Boruta analysis ranked ACAG higher than AG and several conventional indicators. Receiver operating characteristic analysis demonstrated that ACAG provided better discrimination than AG for both outcomes, with the combined Sequential Organ Failure Assessment + ACAG model achieving the best performance (*P* < .01). Associations remained consistent across subgroups without significant interactions. ACAG is a robust independent prognostic marker in septic patients with heart failure, outperforming conventional AG in short-term outcome prediction. Integration of ACAG with Sequential Organ Failure Assessment may enhance early risk stratification and guide clinical decision-making.

## 1. Introduction

The anion gap (AG) is widely used to evaluate metabolic acidosis and to infer the presence of unmeasured anions in critically ill patients. Increases in AG accompany common intensive care unit (ICU) conditions such as lactic acidosis, ketoacidosis, and toxin ingestions, and have been linked to worse outcomes in critical illness.^[1,2]^ However, the AG is strongly influenced by serum albumin. Hypoalbuminemia – prevalent in intensive care – can mask the true elevation of AG and lead to underestimation of metabolic derangements.^[1-3]^

To address this limitation, the albumin-corrected anion gap (ACAG) was proposed, adjusting AG for the prevailing albumin concentration to better reflect the strong-ion and weak-acid contributions to acid–base status.^[3,4]^ Prior work suggests that ACAG improves detection of occult metabolic acidosis and that higher ACAG is associated with mortality across several critically ill populations.^[4-6]^

Sepsis is characterized by profound metabolic and circulatory disturbances and remains a leading driver of ICU admissions and death.^[7]^ Heart failure (HF) is a frequent and clinically important cardiovascular comorbidity in the ICU; in the context of sepsis, HF may exacerbate hemodynamic instability, microcirculatory dysfunction, and tissue hypoperfusion, thereby amplifying metabolic stress and risk of adverse outcomes.^[8,9]^ Whether ACAG provides incremental prognostic information in septic patients with HF has not been fully established.

Accordingly, we aimed to evaluate the prognostic significance of ACAG in adults with sepsis and HF using the Medical Information Mart for Intensive Care IV (MIMIC-IV) database. We hypothesized that ACAG would be independently associated with short-term mortality and would demonstrate superior discrimination compared with conventional AG in this high-risk subgroup.^[10]^

## 2. Materials and methods

### 2.1. Data source and ethical approval

This retrospective cohort study was conducted using data from the Medical Information Mart for Intensive Care IV (MIMIC-IV, version 2.2) database, which contains detailed clinical information of patients admitted to the intensive care units at the Beth Israel Deaconess Medical Center between 2008 and 2019. All data in MIMIC-IV are de-identified, and the use of the database has received approval from the Institutional Review Boards of the Massachusetts Institute of Technology and Beth Israel Deaconess Medical Center. The authors completed the National Institutes of Health training program for human subjects research (certification number: 55981932). Because this study was based on publicly available, de-identified data, informed consent and additional ethical approval were waived.^[11]^

### 2.2. Study population

We included adult patients (≥18 years) who met the diagnostic criteria for sepsis-3, defined as suspected or documented infection accompanied by an acute increase of ≥2 points in the sequential organ failure assessment (SOFA) score.^[7]^ Patients were also required to have a documented diagnosis of heart failure (International Classification of Diseases, ICD-9-CM: 428.x or ICD-10-CM: I50.x). For patients with multiple ICU admissions during the same hospitalization, only the first ICU stay was included.

Exclusion criteria were: estimated glomerular filtration rate < 60 mL/min/1.73 m² or history of chronic dialysis; malignancy or long-term immunosuppressive therapy; missing key variables (anion gap [AG] or serum albumin within 24 hours after ICU admission); lack of 28-day follow-up or in-hospital mortality data; ICU length of stay <24 hours; and concomitant acute coronary syndrome or severe endocrine disorders such as thyroid dysfunction or adrenal insufficiency.

### 2.3. Exposure variable

The ACAG was calculated according to the formula proposed by Figge et al:


$$\begin{array}{l} ACAG=AG+\left(0.25\times \left(44\text{–}albumin\left[g/L\right]\right)\right) \end{array}$$

where 44 g/L represents the reference value for normal serum albumin concentration.^[3,12]^ The conventional AG was calculated as (Na^+^ + K^+^) – (Cl^−^ + HCO_3_^−^). The optimal cutoff point of ACAG for risk stratification was determined using X-tile software (version 3.6.1, Yale University) by maximizing the log-rank *χ*² value, which identified 20 mmol/L as the threshold. Patients were subsequently classified into the low-ACAG group (<20 mmol/L) and high-ACAG group (≥20 mmol/L).

### 2.4. Outcomes

The primary outcomes were 28-day all-cause mortality and in-hospital all-cause mortality. Mortality status was determined from the MIMIC-IV records.

### 2.5. Covariates

Baseline variables extracted within the first 24 hours of ICU admission included demographic information (age, sex), comorbidities, vital signs, laboratory data, urine output, and severity scores (Glasgow Coma Scale, SOFA score, Charlson comorbidity index). For variables with missingness ≤20%, multiple imputation with chained equations (*m* = 10) was applied.

### 2.6. Statistical analysis

Continuous variables are presented as mean ± standard deviation for normally distributed data or median with interquartile range for skewed data, and were compared using Student *t* test or the Mann–Whitney *U* test as appropriate. Categorical variables are expressed as counts (percentages) and compared using the chi-square test or Fisher’s exact test.

Survival differences between groups were assessed using Kaplan–Meier curves with log-rank tests. The independent association between ACAG and outcomes was examined using Cox proportional hazards regression models. Model adjustments were performed sequentially for potential confounders, including age, sex, comorbidities, SOFA score, and key laboratory indicators. Restricted cubic spline regression was employed to explore nonlinear relationships between ACAG and mortality, with knots placed at the 5th, 35th, 65th, and 95th percentiles.

Variable importance for outcome prediction was assessed using the Boruta algorithm based on a random forest classifier.^[13]^ Predictive performance was compared using receiver operating characteristic curves with calculation of the area under the curve (AUC). The DeLong test was used for statistical comparison of AUCs between models.^[14]^ To evaluate incremental predictive value, ACAG and AG were each combined with SOFA score to construct joint models. Subgroup analyses were conducted across age, sex, comorbidities, and disease severity, and interaction terms were tested.

All analyses were performed using R software (version 4.2.0) and Python (version 3.9). Two-sided *P* values < .05 were considered statistically significant.

## 3. Results

### 3.1. Baseline characteristics

A total of 5842 patients with both sepsis and HF were identified from the MIMIC-IV database. After applying exclusion criteria, 715 patients were included in the final analysis, of whom 366 were classified into the high-ACAG group (≥20 mmol/L) and 349 into the low-ACAG group (<20 mmol/L). Baseline characteristics are summarized in Table 1. There were no significant differences in age, sex distribution, or most comorbidities between groups. However, patients in the high-ACAG group had a higher prevalence of uncomplicated diabetes (34.4% vs 26.7%, *P* = .024).

**Table 1:** Baseline characteristics of patients with sepsis and heart failure stratified by albumin-corrected anion gap (ACAG).

Variable	Total (n = 715)	High ACAG (≥20 mmol/L, n = 366)	Low ACAG (<20 mmol/L, n = 349)	Statistic	*P* value
Male sex, n (%)	389 (54.41)	200 (54.64)	189 (54.15)	*χ*² = 0.017	.895
Age, yr, mean ± SD	67.51 ± 15.08	66.45 ± 15.07	68.61 ± 15.04	*t* = −1.915	.056
Cerebrovascular disease, n (%)	103 (14.41)	54 (14.75)	49 (14.04)	*χ*² = 0.074	.786
Chronic pulmonary disease, n (%)	236 (33.01)	112 (30.60)	124 (35.53)	*χ*² = 1.963	.161
Diabetes without complication, n (%)	219 (30.63)	126 (34.43)	93 (26.65)	*χ*² = 5.087	.024
Diabetes with complication, n (%)	47 (6.57)	26 (7.10)	21 (6.02)	*χ*²=0.343	.558
Severe liver disease, n (%)	46 (6.43)	29 (7.92)	17 (4.87)	*χ*² = 2.765	.096
Charlson comorbidity index, median [*Q*1, *Q*3]	6 [4, 7]	6 [4, 7]	6 [4, 7]	*Z* = 1.924	.054
GCS score, mean ± SD	13.58 ± 2.72	13.46 ± 2.91	13.71 ± 2.51	*t* = −1.243	.214
SOFA score, median [*Q*1, *Q*3]	6 [4, 9]	7 [5, 10]	5 [3, 7]	*Z* = 7.431	<.001
Platelets (×10⁹/L), median [*Q*1, *Q*3]	167 [108, 245]	159 [102, 248.75]	171 [121, 240]	*Z* = −1.461	.144
WBC (×10⁹/L), median [*Q*1, *Q*3]	15.60 [10.60, 21.50]	16.75 [11.93, 23.15]	14.30 [9.70, 20.10]	*Z* = 3.764	<.001
BUN (mg/dL), median [*Q*1, *Q*3]	30 [19, 46]	35 [23, 56]	25 [17, 35]	*Z* = 7.422	<.001
Creatinine (mg/dL), median [*Q*1, *Q*3]	1.30 [0.90, 1.90]	1.60 [1.10, 2.40]	1.10 [0.80, 1.40]	*Z* = 9.610	<.001
INR, median [*Q*1, *Q*3]	1.50 [1.22, 2.03]	1.50 [1.30, 2.20]	1.40 [1.20, 1.90]	*Z* = 2.515	.012
PT (s), median [*Q*1, *Q*3]	16.20 [13.80, 21.86]	16.60 [14.20, 23.32]	15.70 [13.53, 20.50]	*Z* = 2.471	.013
ALT (U/L), median [*Q*1, *Q*3]	33 [19, 78]	41 [21, 118]	27 [18, 52]	*Z* = 5.224	<.001
Total bilirubin (mg/dL), median [*Q*1, *Q*3]	0.90 [0.50, 1.80]	1.00 [0.50, 2.10]	0.80 [0.50, 1.40]	*Z* = 2.845	.004
Lactate (mmol/L), median [*Q*1, *Q*3]	2.30 [1.70, 3.40]	2.90 [2.00, 5.00]	1.95 [1.50, 2.50]	*Z* = 10.156	<.001
Arterial pH, mean ± SD	7.30 ± 0.10	7.28 ± 0.12	7.32 ± 0.08	*t* = −6.184	<.001
Heart rate (beats/min), mean ± SD	91.94 ± 17.44	93.97 ± 18.16	89.81 ± 16.41	*t* = 3.213	.001
Mean blood pressure (mm Hg), mean ± SD	74.69 ± 9.54	74.38 ± 9.27	75.01 ± 9.82	*t* = −0.874	.383
Respiratory rate (/min), mean ± SD	21.61 ± 4.30	22.23 ± 4.52	20.96 ± 3.96	*t* = 3.993	<.001
Temperature (°C), mean ± SD	37.61 ± 0.85	37.61 ± 0.86	37.60 ± 0.84	*t* = 0.070	.944
SpO₂ (%), mean ± SD	89.92 ± 7.82	89.55 ± 8.77	90.32 ± 6.67	*t* = −1.327	.185
Glucose (mg/dL), median [*Q*1, *Q*3]	168 [130.50, 228.50]	182 [140.25, 259.25]	159 [124, 204]	*Z* = 5.092	<.001
Urine output (mL), median [*Q*1, *Q*3]	1395 [784, 2335]	1237.50 [655, 2133.75]	1570 [996, 2550]	*Z* = −4.088	<.001
28-d mortality, n (%)	166 (23.22)	115 (31.42)	51 (14.61)	*χ*² = 28.310	<.001
In-hospital mortality, n (%)	155 (21.68)	109 (29.78)	46 (13.18)	*χ*² = 28.997	<.001
AG (mmol/L), mean ± SD	18.45 ± 5.31	21.87 ± 5.15	14.87 ± 2.19	*t* = 23.828	<.001
ACAG (mmol/L), mean ± SD	21.08 ± 5.34	24.72 ± 5.04	17.26 ± 1.79	*t* = 26.597	<.001

Values are presented as mean ± SD, median [*Q*1, *Q*3], or n (%), as appropriate. Comparisons between groups were performed using independent-samples *t* test, Mann–Whitney *U* test, *χ*² test, or Fisher exact test as applicable.

ACAG = albumin-corrected anion gap, AG = anion gap, ALT = alanine aminotransferase, BUN = blood urea nitrogen, GCS = Glasgow Coma Scale, INR = international normalized ratio, PT = prothrombin time, SD = standard deviation, SOFA = sequential organ failure assessment, SpO₂ = peripheral oxygen saturation, WBC = white blood cell count.

With respect to disease severity and laboratory findings, patients in the high-ACAG group had significantly higher SOFA scores, white blood cell counts, blood urea nitrogen, and serum creatinine levels (all *P* < .001). Heart rate and respiratory rate were also higher in the high-ACAG group. In contrast, urine output was lower compared with the low-ACAG group. Notably, both 28-day and in-hospital mortality were significantly increased in the high-ACAG group (31.4% vs 14.6% and 29.8% vs 13.2%, respectively; both *P* < .001).

### 3.2. Kaplan–Meier survival and Cox regression analyses

**Figure 1.**
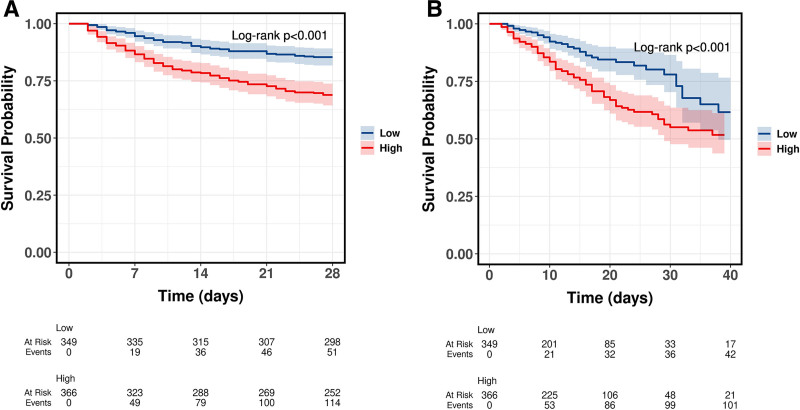
Kaplans–Meier survival curves for (A) 28-day and (B) in-hospital mortality in patients with sepsis and heart failure, stratified by albumin-corrected anion gap (ACAG). Patients in the high-ACAG group (≥20 mmol/L) had significantly lower survival probabilities than those in the low-ACAG group (<20 mmol/L; log-rank *P* < .001). ACAG = albumin-corrected anion gap.

Kaplan–Meier curves demonstrated significantly lower survival probabilities in the high-ACAG group for both 28-day and in-hospital mortality (log-rank *P* < .001; Fig. 1). In unadjusted Cox regression, high ACAG was associated with increased risks of 28-day (hazard ratio [HR] = 2.36, 95% confidence interval [CI]: 1.70–3.29, *P* < .001) and in-hospital mortality (HR = 1.96, 95% CI: 1.39–2.77, *P* < .001). These associations remained significant after adjustment for age, sex, SOFA score, Charlson comorbidity index, and laboratory variables (28-day mortality: HR = 1.80, 95% CI: 1.27–2.56, *P* = .001; in-hospital mortality: HR = 1.75, 95% CI: 1.20–2.54, *P* = .003; Table 2).

**Table 2:** Association between albumin-corrected anion gap (ACAG) and mortality in univariable and multivariable Cox regression analyses.

Outcome	Group	β	SE	Z	*P* value	HR (95% CI)	β (adjusted)	SE	Z	*P* value	HR (95% CI)
28-d mortality	Low ACAG	–	–	–	–	1.00 (Reference)	–	–	–	–	1.00 (Reference)
	High ACAG	0.86	0.17	5.10	<.001	2.36 (1.70–3.29)	0.59	0.18	3.28	<.001	1.80 (1.27–2.56)
In-hospital mortality	Low ACAG	–	–	–	–	1.00 (Reference)	–	–	–	–	1.00 (Reference)
	High ACAG	0.67	0.18	3.81	<.001	1.96 (1.39–2.77)	0.56	0.19	2.93	.003	1.75 (1.20–2.54)

Cox proportional hazards regression was used. Univariable and multivariable hazard ratios (HRs) are shown. Multivariable models were adjusted for baseline demographics, comorbidities, SOFA score, and laboratory variables.

ACAG = albumin-corrected anion gap, CI = confidence interval, HR = hazard ratio, SE = standard error.

### 3.3. Restricted cubic spline analysis

**Figure 2.**
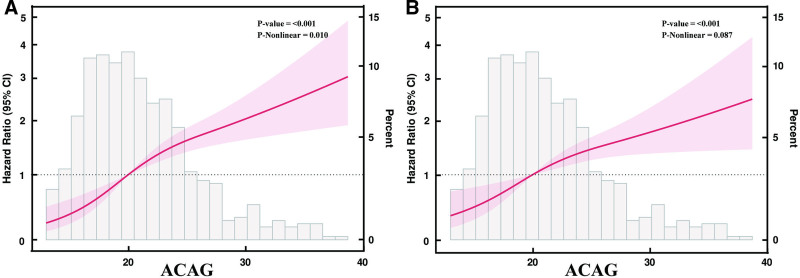
Restricted cubic spline (RCS) analysis showing the association between ACAG and (A) 28-day and (B) in-hospital mortality. The hazard ratio (HR) increases with higher ACAG, with evidence of nonlinearity for 28-day mortality (P for nonlinearity = 0.010) but not for in-hospital mortality (P for nonlinearity = 0.087). ACAG = albumin-corrected anion gap, CI = confidence interval, HR = hazard ratio, RCS = restricted cubic spline.

Restricted cubic spline models revealed that ACAG was positively associated with 28-day mortality in a nonlinear fashion (overall *P* < .001; *P* for nonlinearity = .010; Fig. 2A). For in-hospital mortality, the association was also positive (overall *P* < .001), but the trend was approximately linear (*P* for nonlinearity = .087; *P* for linearity < .001; Fig. 2B).

### 3.4. Variable importance ranking

**Figure 3.**
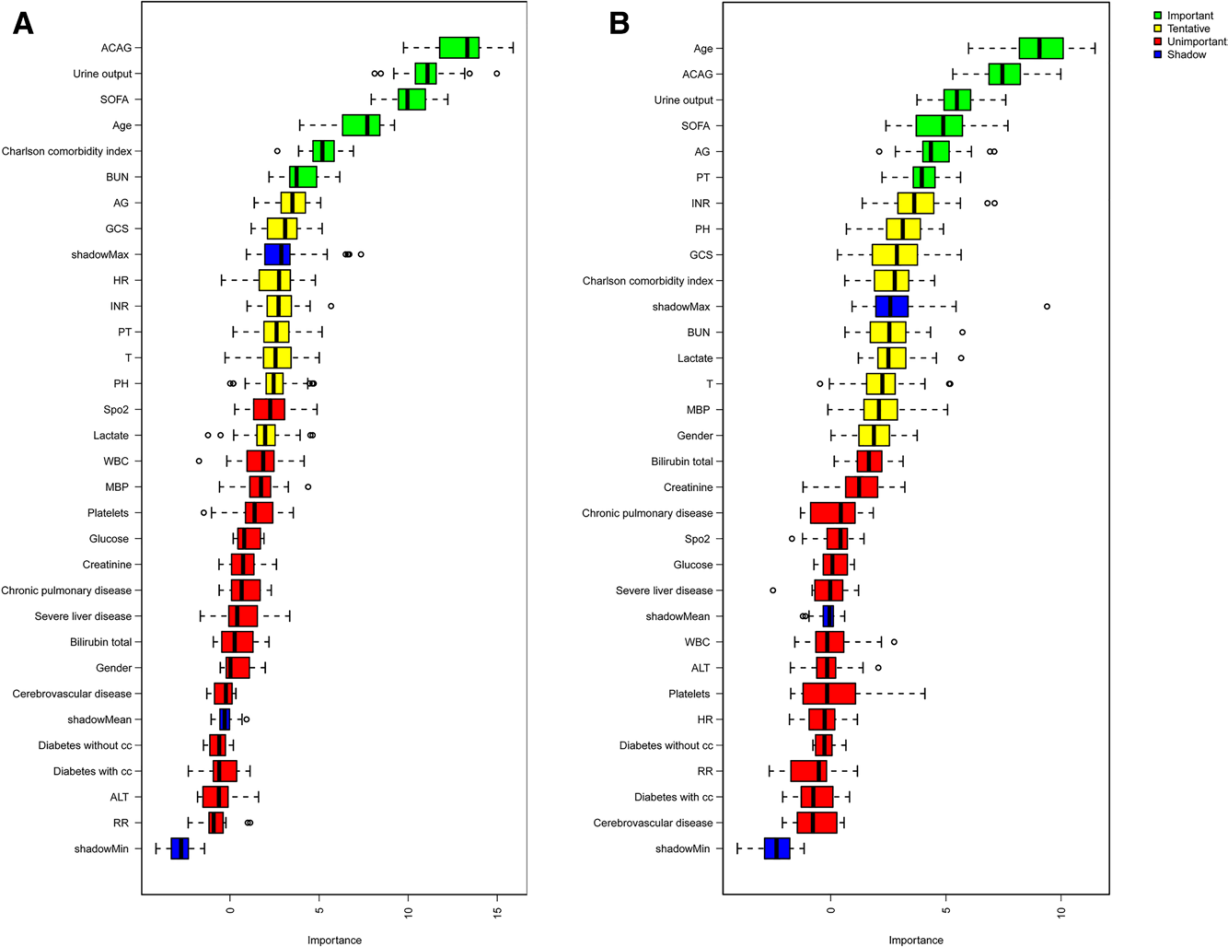
Variable importance ranking using the Boruta feature selection algorithm for (A) 28-day and (B) in-hospital mortality. ACAG showed higher predictive importance than conventional anion gap (AG) and several other clinical indicators. ACAG = albumin-corrected anion gap, AG = anion gap, ALT = alanine aminotransferase, BUN = blood urea nitrogen, HR = heart rate, INR = international normalized ratio, MBP = mean blood pressure, PT = prothrombin time, RR = respiratory rate, SOFA = sequential organ failure assessment, T = temperature, pH = arterial pH, WBC = white blood cell count.

Using the Boruta feature-selection algorithm, ACAG was retained as a confirmed important feature and ranked among the top predictors for both 28-day and in-hospital mortality – consistently above AG, lactate and comparable to or higher than urine output and the SOFA score (Fig. 3).

### 3.5. Discrimination performance

**Figure 4.**
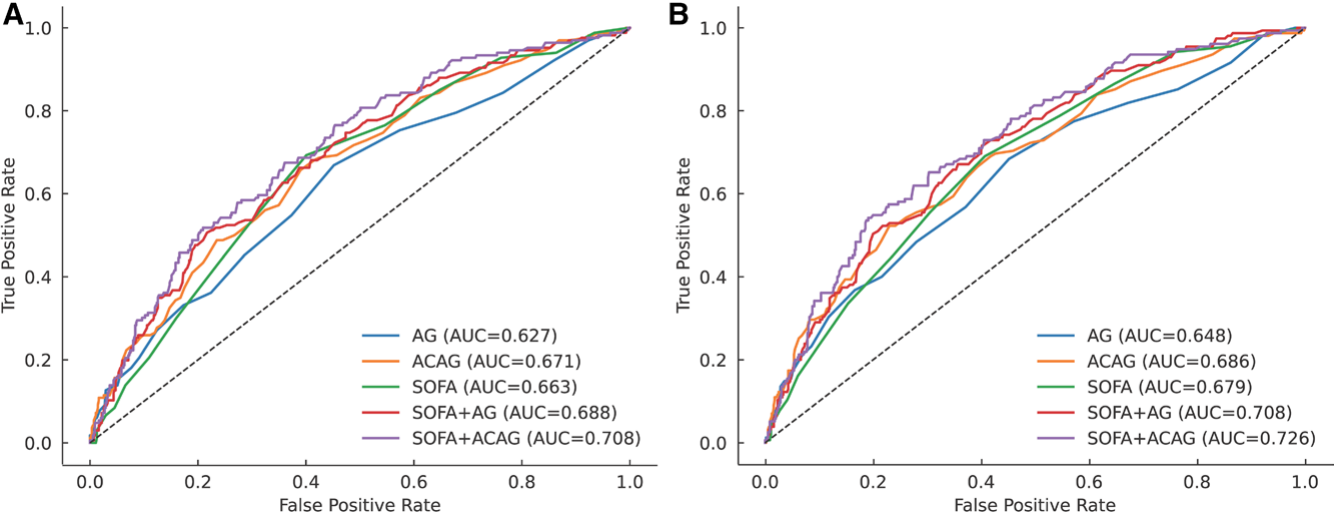
Receiver operating characteristic (ROC) curves for predicting (A) 28-day and (B) in-hospital mortality. ACAG demonstrated higher discrimination compared with AG, and the SOFA + ACAG model achieved the best performance. ACAG = albumin-corrected anion gap; AG = anion gap, AUC = area under the curve, ROC = receiver operating characteristic, SOFA = sequential organ failure assessment.

Receiver operating characteristic analysis showed that ACAG yielded higher AUC values than AG for both 28-day mortality (0.671 vs 0.627, *P* < .001) and in-hospital mortality (0.686 vs 0.648, *P* = .0004). When combined with SOFA score, the SOFA + ACAG model achieved the best discrimination (28-day mortality: AUC = 0.709; in-hospital mortality: AUC = 0.726), significantly outperforming SOFA alone and SOFA + AG models (all *P* < .01; Fig. 4, Table 3).

**Table 3:** Comparison of area under the curve (AUC) values between models using the DeLong test.

Outcome	Model 1	Model 2	ΔAUC	SE	*Z*	*P* value
28-d mortality	AG	ACAG	−0.0441	0.0094	−4.683	<.0001
In-hospital mortality	AG	ACAG	−0.0383	0.0108	−3.537	.0004
28-d mortality	SOFA + AG	SOFA + ACAG	−0.0208	0.0055	−3.787	.0002
In-hospital mortality	SOFA + AG	SOFA + ACAG	−0.0186	0.0059	−3.153	.0016

Cox proportional hazards regression was used. Univariable and multivariable hazard ratios (HRs) are shown. Multivariable models were adjusted for baseline demographics, comorbidities, SOFA score, and laboratory variables.

ACAG = albumin-corrected anion gap, CI = confidence interval, HR = hazard ratio, SE = standard error.

### 3.6. Subgroup analysis

**Figure 5.**
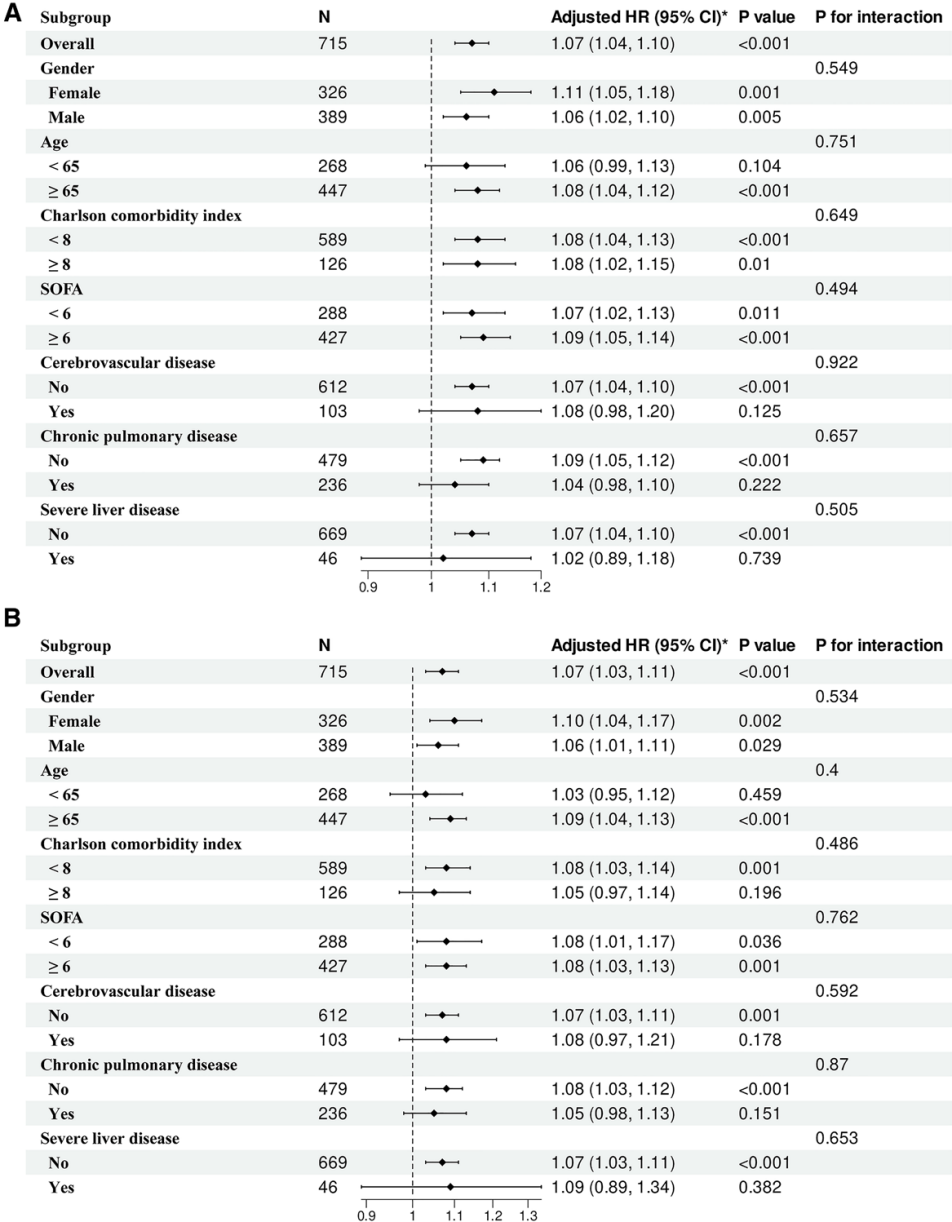
Subgroup analysis of the association between high ACAG (≥20 mmol/L) and mortality, shown as hazard ratios (HRs) with 95% confidence intervals (CIs). Forest plots display results for (A) 28-day and (B) in-hospital mortality across predefined subgroups. No significant interactions were observed. ACAG = albumin-corrected anion gap, CI = confidence interval, HR = hazard ratio, SOFA = sequential organ failure assessment.

Subgroup analyses showed consistent associations between high ACAG and increased mortality across age groups, sex, Charlson comorbidity index strata, SOFA score categories, and major comorbidities including cerebrovascular disease, chronic pulmonary disease, and severe liver disease. No significant interactions were observed (all *P* for interaction > .05; Fig. 5), indicating the robustness of the findings.

## 4. Discussion

In this large retrospective cohort of critically ill patients with sepsis and HF, we found that elevated ACAG was independently associated with both 28-day and in-hospital mortality. Compared with conventional AG, ACAG demonstrated stronger predictive performance, and its combination with SOFA score further improved prognostic discrimination. Restricted cubic spline analysis indicated a nonlinear relationship between ACAG and short-term mortality, and the association was robust across clinically relevant subgroups. These findings suggest that ACAG is a valuable metabolic marker for early risk stratification in this high-risk population.

The prognostic significance of ACAG may be explained by its ability to capture unmeasured anions and metabolic stress more accurately than AG, particularly in the setting of hypoalbuminemia. Hypoalbuminemia is highly prevalent in sepsis and HF due to systemic inflammation, impaired hepatic synthesis, and nutritional depletion.^[15,16]^ In such patients, conventional AG may underestimate the severity of metabolic derangements, whereas ACAG adjusts for albumin levels and better reflects the true burden of retained anions such as lactate, phosphate, and organic acids.^[3,6]^ Consistent with expectations, patients with higher ACAG also exhibited higher lactate. However, in this study ACAG is primarily intended to index the burden of unmeasured anions after correcting for hypoalbuminemia – that is an enhanced acid–base marker – rather than a substitute for lactate or a direct surrogate of tissue hypoperfusion or cardiac dysfunction.^[1-4]^

In addition, elevated ACAG in septic patients with HF may reflect multiple overlapping mechanisms. Septic cardiomyopathy, characterized by impaired myocardial contractility and reduced perfusion, contributes to lactate accumulation and excess unmeasured anions.^[8,17]^ Classic studies have also shown that septic shock can lead to profound but reversible myocardial depression.^[18]^ Hepatic congestion in HF impairs the clearance of organic acids and bilirubin-related metabolites, further elevating the corrected AG.^[19]^ Renal dysfunction, common in both sepsis and HF, diminishes the excretion of fixed acids and promotes acidosis.^[20]^ Moreover, systemic inflammation and hypoalbuminemia alter the distribution of strong ions, amplifying the corrected AG and reflecting immune–metabolic crosstalk in HF.^[21-23]^ Collectively, these mechanisms suggest that ACAG should be interpreted as an integrative marker of metabolic stress rather than a direct causal factor.

Our results extend prior observations in heterogeneous ICU cohorts by focusing specifically on septic patients with HF. Previous studies have shown that ACAG outperforms AG in predicting mortality among critically ill patients with acute kidney injury or advanced age.^[5,8,20]^ Moreover, studies in acute myocardial infarction and chronic kidney disease populations have also demonstrated that elevated ACAG is associated with increased mortality risk.^[15,19]^ However, evidence in patients with combined sepsis and HF has been scarce. This group represents a particularly vulnerable population, as sepsis-induced systemic inflammation and microcirculatory dysfunction are compounded by impaired cardiac output and venous congestion in HF, leading to profound metabolic stress.^[21,22,24]^ Our study provides novel evidence that ACAG is independently predictive in this subgroup and may reflect overlapping pathways of metabolic acidosis, tissue hypoxia, and organ dysfunction.

### 4.1. Clinical implications

The clinical implications of these findings are noteworthy. As a routinely available and inexpensive laboratory measure, ACAG can be calculated at the bedside within minutes and offers incremental prognostic information beyond conventional scores such as SOFA. Conceptually, ACAG is an albumin-adjusted refinement of the traditional AG that better indexes the burden of unmeasured anions in the setting of hypoalbuminemia. Identifying septic patients with HF who present with high ACAG may facilitate earlier recognition of those at greatest risk and prompt targeted monitoring and management (e.g., tighter fluid balance, individualized vasopressor strategies, and metabolic support). Future studies should test ACAG-informed care pathways that integrate ACAG with established biomarkers (e.g., lactate, BNP/NT-proBNP) and selected echocardiographic/hemodynamic assessments to determine whether such strategies translate into improved outcomes.^[25]^

### 4.2. Limitations

Several limitations should be acknowledged. First, this was a retrospective analysis from a single database (MIMIC-IV), and residual confounding cannot be excluded; the generalizability and transportability of the findings require external validation in multi-institutional and prospective cohorts. Second, echocardiographic and advanced hemodynamic variables (e.g., LVEF, diastolic indices, CO/CI, CVP, SvO₂) were incompletely and non-uniformly available, so we did not examine direct correlations between ACAG and those measures.^[26]^ We also did not perform head-to-head comparisons with BNP/NT-proBNP because these measurements were not uniformly available around ICU admission in MIMIC-IV. Third, although major comorbidities and laboratory covariates were adjusted for, unmeasured factors (e.g., nutritional status, dynamic liver/kidney function changes) may still have influenced both ACAG and outcomes. Feature-importance findings from Boruta should be interpreted as complementary to, rather than a substitute for, formal head-to-head discrimination analyses. Fourth, ACAG is calculated using standard formulas and laboratory measurements that may vary across clinical contexts and sampling times. Finally, the X-tile–derived threshold is cohort-specific and requires external validation, and causal inference cannot be drawn from observational data. Notwithstanding these caveats, strengths include use of a large, well-curated critical-care dataset, prespecified and comprehensive statistical analyses (including spline modeling and feature selection), and robust, directionally consistent associations across outcomes and clinically relevant subgroups.

## 5. Conclusion

In conclusion, ACAG is an independent and robust prognostic indicator in septic patients with HF, outperforming conventional AG in predicting short-term mortality. Interpreted within the acid–base framework as an albumin-adjusted index of unmeasured anion burden, ACAG complements established tools such as SOFA and biochemical markers (e.g., lactate, BNP/NT-proBNP) and may help refine early risk stratification and individualized management in this high-risk population. Future work should assess the generalizability of ACAG in multi-institutional, prospective cohorts and evaluate integrated strategies that combine ACAG with echocardiography and invasive hemodynamic assessments.^[27]^

## Author contributions

**Conceptualization:** Qiao Xiao, Zhiling Sun.

**Data curation:** Tingting Liu, Xinyuan Zhang.

**Formal analysis:** Qiao Xiao, Tingting Liu.

**Methodology:** Xinyuan Zhang.

**Supervision:** Zhiling Sun.

**Writing – original draft:** Qiao Xiao.

**Writing – review & editing:** Tingting Liu, Xinyuan Zhang, Zhiling Sun.
